# Improving Brain Metastases Outcomes Through Therapeutic Synergy Between Stereotactic Radiosurgery and Targeted Cancer Therapies

**DOI:** 10.3389/fonc.2022.854402

**Published:** 2022-03-02

**Authors:** Sebastian Rubino, Daniel E. Oliver, Nam D. Tran, Michael A. Vogelbaum, Peter A. Forsyth, Hsiang-Hsuan Michael Yu, Kamran Ahmed, Arnold B. Etame

**Affiliations:** ^1^ Department of Neuro-Oncology, Moffitt Cancer Center, Tampa, FL, United States; ^2^ Department of Radiation Oncology, Moffitt Cancer Center, Tampa, FL, United States

**Keywords:** targeted therapy, SRS, brain metastases, non-small cell lung cancer, breast cancer, melanoma, renal cell carcinoma

## Abstract

Brain metastases are the most common form of brain cancer. Increasing knowledge of primary tumor biology, actionable molecular targets and continued improvements in systemic and radiotherapy regimens have helped improve survival but necessitate multidisciplinary collaboration between neurosurgical, medical and radiation oncologists. In this review, we will discuss the advances of targeted therapies to date and discuss findings of studies investigating the synergy between these therapies and stereotactic radiosurgery for non-small cell lung cancer, breast cancer, melanoma, and renal cell carcinoma brain metastases.

## Introduction

Brain metastases are the most common malignant tumors found in the central nervous system (1). They are 10 times more common than primary central nervous system (CNS) brain tumors, affecting 20 to 40% of all patients with cancer, and greater than 100,000 new patients each year in the United States (2–4). With improved therapies, increased screening of neurologically asymptomatic patients, and patients living longer, the incidence of brain metastases continues to increase. The blood-brain barrier has long posed a challenge for traditional chemotherapeutics to enter the brain and effectively treat these lesions. Therefore, the mainstays of treatment, to date, have included surgery, stereotactic radiosurgery (SRS), and whole brain radiotherapy; with only a limited role for systemic therapies (5).

The current treatment algorithm for patients with brain metastases includes stratification by symptoms, as well as disease burden by number (single lesion, oligometastases, polymetastases) and size (6, 7). Symptomatic patients with poor performance status often benefit from best supportive care alone (8). Symptomatic patients with a favorable performance status may be candidates for surgery and/or radiotherapy (SRS, hypofractionated radiosurgery, or whole brain radiotherapy) depending on the number and size of the metastases, in addition to treatment with systemic therapy (either traditional chemotherapies, immunotherapies and/or targeted molecular therapies depending on the molecular signature of the primary tumor) (9). Asymptomatic patients with small lesions may be treated with upfront systemic therapy, while saving radiotherapy and/or neurosurgery as salvage therapy (5).

Increasing knowledge of primary tumor biology, actionable molecular targets and continued improvements in systemic and radiotherapy regimens have helped improve survival but necessitates multidisciplinary collaboration between neurosurgical, medical and radiation oncologists. In this review, we will discuss the advances of targeted therapies to date and discuss findings of studies investigating the synergy between these therapies and SRS for the treatment of non-small cell lung cancer, breast cancer, melanoma, and renal cell carcinoma brain metastases.

### Non-Small Cell Lung Cancer

Non-small cell lung cancer (NSCLC) accounts for approximately 85% of all lung cancers, with 16 to 34% of all NSCLC patients experiencing brain metastases and 40 to 50% of all patients with brain metastases having lung etiology (10–13). With evolution of targeted therapies, molecular testing for the following oncogenic driver mutations has become standard of care; *ALK* (Anaplastic lymphoma kinase) rearrangements, *BRAF* (B-Raf proto-oncogene, serine/threonine kinase) mutations, *EGFR* (epidermal growth factor receptor) mutations, *MET* (mesenchymal–epithelial transition) exon 14 skipping mutations, *NTRK* (Neurotrophic Tyrosine Receptor Kinase) 1/2/3 gene fusions, *RET* (ret proto-oncogene) rearrangements, and *ROS1* (c-ros oncogene 1) rearrangements (14–16). Mutations in *EGFR, ALK, BRAF, NTRK, MET, RET, ROS1, KRAS* (Kirsten rat sarcoma virus)*, HER2* (human epidermal growth factor receptor 2) genes have all been found to be expressed in NSCLC and have targeted therapies inhibiting the abnormal proteins for which these mutated genes encode. First-generation EGFR inhibitors, erlotinib and gefitinib, and second-generation EGFR ErbB family inhibitor, afatinib, have been replaced by third generation EGFR tyrosine kinase inhibitor osimertinib as first-line therapy in patients with EGFR-mutated brain metastases secondary to improved CNS penetration, efficacy, longer response and survival duration (13, 17, 18). Alectinib, brigatinib, and loratinib are preferred first-line agents for patients with brain metastases containing *ALK* rearrangements (19–21). Selpercatinib (22) and pralsetinib (23) are selective RET inhibitors that are used in the treatment of patients with RET fusion-positive NSCLC, while Entrectinib (24) is a ROS1 fusion inhibitor used in the treatment of ROS1 fusion-positive NSCLC.

The individual efficacies of SRS and targeted therapies for NSCLC have led many to investigate the synergy between these two therapies and to investigate how it can best be maximized (**Table 1**). A retrospective study in 2018 by Yomo et al. assessed 133 patients with brain metastases arising from EGFR-mutant lung adenocarcinoma who received upfront gamma knife SRS and subsequently were administered EGFR tyrosine kinase inhibitors. 1-year and 2-year overall survival rates were 74 and 52%, respectively with a mean survival time of 24.8 months (27). These outcomes are significantly better than prior studies and showed median survival time from initial brain metastases treatment rose from 7 months between 1985 and 2005 to 12 months between 2006 and 2014 (29). The Oda study also reported 1-year and 2-year distant brain metastases recurrence rates were 34 and 53% respectively, and 1- and 2-year local tumor control per lesion were 97% and 95%, respectively. Multivariate analysis showed that being EGFR tyrosine kinase naïve was associated with longer overall survival (HR: 0.42, P < 0.001), a lower distant intracranial recurrence rate (HR: 0.61, P=0.037), and a higher local tumor control rate (HR: 0.28, P=0.001) (27). The underpinnings of synergy between SRS and targeted therapies are highlighted by these findings. The lower distant intracranial recurrence rate indicates that the targeted therapies help to address metastases beyond the SRS field, while the higher local tumor control rate may be theorized to occur secondary to improved breakdown of the blood-brain barrier within the SRS bed, therefore lending to increased efficacy of the targeted therapies in these regions.

**Table 1:** Studies evaluating synergy between SRS and targeted therapies in patients with NSCLC brain metastases (BrM).

Study Identifier	Study Period	Study Size (n = patients)	Treatment/Intervention Groups	Results	References
Magnusen et al.	2008-2014	N = 351	Patients with EGFR-mutant NSCLC BrM treated with SRS followed by EGFR-tyrosine kinase inhibitor (TKI) *vs* WBRT followed by EGFR-TKI *vs* EGFR-TKI followed by SRS or WBRT at intracranial progression	• Median OS for SRS followed by EGFR-TKI, WBRT followed by TKI and EGFR-TKI followed by SRS or WBRT = 46, 30, and 25 months respectively (p < .001) • On MVA, SRS versus EGFR-TKI, WBRT versus EGFR-TKI, age, performance status, EGFR exon 19 mutation, and absence of extracranial metastases associated with improved OS	(25)
Moraes et al.	2008-2017	N = 218	Patients with EGFRm and EGFRwt NSCLC BrM treated with SRS ± systemic therapy (chemotherapy, TKI or immunotherapy)	• 24-month incidence of LF was 6% and 16% for EGFRm BrM and EGFRwt, respectively (0.43 (0.19-0.95); p = 0.037) • 24-month incidence of RN was 4% and 6% for EGFRm and EGFRwt BrM, respectively (0.8 (0.32-1.98) p = 0.63) • On MVA, BrM size and prescription dose (PD) significantly correlated with a higher risk of LF and BrM size correlated with a higher risk of RN	(26)
Yomo et al	2010-2016	N = 133	Patients with EGFR-mutant lung adenocarcinoma BrM who received upfront Gamma Knife SRS; post-SRS EGFR-TKI administered to 85% of cohort	• 1-year OS = 74%, 2-year OS 52% • 1-year and 2-year distant BrM recurrence rates (per patient) after SRS = 34% and 53% • 1-year and 2-year rates of local tumor control (per lesion) = 97% and 95% • MVA proportional hazards analyses found being EGFR-TKI naïve • associated with longer OS (HR: 0.42, P < 0.001), a lower distant intracranial recurrence rate (HR: 0.61, P = 0.037) and higher local tumor control rate (HR: 0.28, P = 0.001)	(27)
Dohm et al	2015-2019	N = 174	Patients with NSCLC BrM treated with single-fraction SRS sessions within 3 months of receiving immune checkpoint inhibitors (ICI), EGFR-tyrosine kinase inhibitors (TKI), chemotherapy and ICI, or standard chemotherapy	• 12-month DIC was 35%, 53%, 41%, and 20% (P = 0.02) for ICI, EGFR-TKI, ICI and chemotherapy, and chemotherapy alone groups, respectively • No differences were noted in LC (P = 0.1) and OS (P = 0.5) between treatment groups • On MVA, factors found to be significant for improved DIC included treatment with EGFR-TKI therapy compared to conventional chemotherapy (HR 0.4; 95% CI 0.25-0.76; P = 0.04) and treatment with SRS before systemic therapy (HR 0.6; 95% CI 0.3-0.9; P = 0.03) • On MVA, factors found to be significant for improved LC included treatment with SRS before (HR 0.4; 95% CI 0.2-0.9; P = 0.03) or concurrent (HR 0.3; 95% CI 0.1-0.6; P = 0.003) compared to following receipt of systemic therapy • Rates of radiation necrosis (RN) did not differ between treatment groups	(28)

Magnuson et al. conducted a multi-institutional retrospective pooled analysis of 351 patients with EGFR-mutant NSCLC-developed brain metastases who received SRS followed by EGFR-TKI, WBRT followed by EGFR-TKI, or EGFR-TKI followed by SRS or WBRT at intracranial progression and found best overall survival times in patients who received SRS followed by EGFR-TKI compared to those who received whole brain radiotherapy first followed by EGFR tyrosine kinase inhibitors and to those who received EGFR tyrosine kinase inhibitor treatment first followed by SRS or whole brain radiotherapy (25). SRS followed by EGFR-TKI resulted in the longest median survival time of 46 months while avoiding the neurocognitive impairment associated with whole brain radiotherapy.

A recent analysis of a prospective registry of 218 patients with NSCLC EGFR-mutated (EGFRm) and EGFR-wild-type brain metastases treated with SRS plus or minus systemic therapies, did not show a statistically significant difference in local failure or radionecrosis rate at 24 months in EGFRm patients with administration of tyrosine kinase inhibitor before SRS (3% and 3%) or after SRS (17% and 0%). Although not reaching statistical significance, receiving TKI before SRS led to a 3% local failure rate of 24 months compared to 17% when administered after SRS (26). The authors did not ascribe these results to lack of synergy but rather concluded that this highlights the importance of not delaying the initiation of systemic therapy with tyrosine kinase inhibitors. On multivariate analysis, brain metastases size and dose of radiation significantly correlated with a higher risk of local failure and brain metastases size correlated with a higher risk of radiation necrosis.

A retrospective study by Dohm et al. of 174 NSCLC brain metastases patients treated with SRS within 3 months of receiving systemic therapies found significantly improved distant intracranial control with EGFR-TKI therapy compared to conventional chemotherapy (HR 0.4; 95% CI 0.25-0.76; P = 0.04) and with receiving SRS before systemic therapy (HR 0.6; 95% CI 0.3-0.9; P = 0.03) (28). Local control was found to be significantly improved when patients received treatment with SRS before (HR 0.4; 95% CI 0.2-0.9; P = 0.03) or concurrent (HR 0.3; 95% CI 0.1-0.6; P = 0.003) with the receipt of systemic therapy. These findings are consistent with immunotherapy literature reporting improved distant brain control for patients receiving stereotactic radiation during or prior to anti-PD1/PD L1 therapy (30, 31).

### Breast Cancer

Breast cancer is the most common malignancy and second leading cause of cancer-related death in women in the United States (32) and the second most common pathology that metastasizes to the brain after lung cancer overall (33, 34). Approximately 10 to 30% of patients with breast cancer will develop brain metastases (35–37). The heterogeneity of breast cancer necessitates a multidisciplinary approach because there are a multitude of systemic therapies that vary depending on the type of breast cancer. There are 5 main types of breast cancer, which include luminal A (hormone receptor positive, HER-2 low expression, with low levels of Ki-67), luminal B (hormone receptor positive, either HER-2 positive or low expression, with high levels of Ki-67), HER-2 positive (hormone receptor low expression), triple negative (hormone receptor and HER-2 low expression) and finally normal breast-like (38).

Most of the literature investigating the synergy between SRS and breast cancer metastases involves HER-2 positive breast cancer brain metastases (**Table 2**). HER-2 is a member of the transmembrane tyrosine kinase EGFR family. It is overexpressed in approximately 14% of breast cancers but has a high incidence of brain metastases and accounts for approximately 44% of resected breast cancer brain metastases (45–47). Trastuzumab was the first anti-HER-2 antibody proved to enhance extracranial disease control and survival rates in patients with metastatic HER-2 positive breast cancer (48). This was followed by trastuzumab emtansine (T-DM1) which uses the trastuzumab antibody to deliver emtansine (DM1) to HER2 antigen expressing tumors (49–52). Unfortunately, several studies have shown the utilization of T-DM1 with SRS significantly increases symptomatic radiation necrosis rates when used concurrently or sequentially. For this reason, utilization of this drug decreased given that SRS is a mainstay of therapy for patients with brain metastases. However, a recent study by Mills at al reporting on a single institution series of 16 patients with HER-2 positive breast cancer who underwent SRS and T-DM1 therapy delivered within 6 months showed only 1 case (3%) of symptomatic radionecrosis (39). The authors hypothesized that those prior studies showing increased rates of radionecrosis included longer time intervals from radiation and potentially do not accurately reflect toxicity from the combined treatment. Furthermore, longer survival may also confound the incidence of radiation necrosis, which may not necessarily be caused by late toxicity of concurrent SRS and T-DM1 administration.

**Table 2:** Studies evaluating synergy between SRS and targeted therapies in patients with breast cancer brain metastases (BrM).

Study Identifier	Study Period	Study Size (n = patients)	Treatment/Intervention Groups	Results	References
Mills et al.	2013-2019	N = 16	Patients with HER2+ breast cancer BrM treated with SRS/FSRT with T-DM1 delivered within 6 months	• Stereotactic radiation delivered concurrently with T-DM1 in (48%) • 1-year LC, DIC, systemic PFS, and OS were 75, 50, 30, and 67%, respectively • 1 case of leptomeningeal progression and 1 case (3%) of symptomatic radionecrosis	(39)
Gori et al.	2005-2014	N= 154	Patients with HER2+ breast cancer BrM treated with local and systemic therapies	• Median OS = 24.5 months • Patients receiving surgery/SRS experienced longer OS compared to those receiving whole-brain radiotherapy or no treatment (33.5 *vs*. 11.4 months; P < .001) • WBRT did not improve OS compared to no treatment (11.4 *vs*. 9.8 months; p = .99) • HER2-targeted therapy was associated with better OS compared to systemic therapy without HER2-targeted therapy or no systemic therapy (27.5 *vs*. 5.4 months; P < .001) • On MVA stratified by local treatments, systemic therapy, KPS, and neurologic symptoms significantly affected OS	(40)
Miller et al.	1998-2014	N = 547	Patients with different molecular subtypes of breast cancer and BrM treated with radiotherapy +/- targeted therapies	• Median OS = significantly shorter in the basal cohort (8.4 months) and progressively increased in luminal A (12.3 months), HER2-positive (15.4 months), and luminal B (18.8 months) cohorts (P < .001) • Among patients with HER2-amplified disease, the median OS increased with use of both HER2 antibodies (17.9 months *vs* 15.1 months; P5.04) and TKIs (21.1 months *vs* 15.4 months; P=.03) • 12-month cumulative incidences of local failure among molecular subtypes were 6.0% in the luminal A cohort, 10.3% in the luminal B cohort, 15.4% in the HER2-positive cohort, and 9.9% in the basal cohort (P = .01) • Concurrent HER2/EGFR TKIs with SRS significantly decreased the 12-month cumulative incidence of local failure from 15.1% to 5.7% (P < .001)	(41)
Kim et al.	2005-2014	N = 84	Patients with newly diagnosed HER2+ breast cancer BrM who treated with SRS and divided into 2 cohorts based on timing of treatment with lapatinib	• 132 lesions (27%) treated with SRS + concurrent lapatinib, 355 (73%) treated with SRS alone. • SRS + concurrent lapatinib group had higher rates of complete response (35% *vs* 11%, P = 0.008) • Per-lesion basis, best objective response superior in SRS + concurrent lapatinib group (median 100% *vs* 70% reduction, P < 0.001) • SRS + concurrent lapatinib group not associated with increased risk of grade 2+ RN (1.0% *vs* 3.5% without, P = 0.27)	(42)
Parsai et al.	1997-2015	N = 126	Patients with HER2+ breast cancer BrM who underwent treatment with lapatinib and SRS	• Concurrent lapatinib was associated with reduction in local failure at 12 months (5.7% *vs* 15.1%, p < 0.01) • For lesions ≤ 75th percentile by volume, concurrent lapatinib significantly decreased local failure • Any use of lapatinib after development of brain metastasis improved median survival compared to SRS without lapatinib (27.3 *vs* 19.5 months, p = 0.03) • 12-month risk of RN was consistently lower in the lapatinib cohort compared to the SRS-alone cohort (1.3% *vs* 6.3%, p < 0.01), despite extended survival	(43)
Figura et al.	2015-2018	N = 15	Patients who received stereotactic radiotherapy for HR+ BrM within 6 months of CDK4/6 inhibitor administration; RT was delivered concurrently, before, or after CDK4/6 inhibitors in 18 (43%), 9 (21%), and 15 (36%) lesions, respectively	• 6- and 12-month local control of treated lesions = 88% and 88%, respectively • 6- and 12-month distant brain control = 61% and 39%, respectively • Median OS was 36.7 months from the date of BrM diagnosis	(44)

The Italian HERBA trial retrospectively evaluated 154 patients across 14 institutions and reported longer overall survival in patients receiving surgery/SRS (33.5 *vs*. 11.4 months for patients receiving WBRT or no treatment; HR = 0.34; 95% confidence interval, 0.22-0.52; P <.001) and in patients receiving HER-2 targeted therapies (27.5 *vs*. 5.4 months in patients receiving non-HER2-targeted therapy or no systemic therapy; HR = .26; 95% confidence interval, 0.17-0.41; P <.001) (40). However, this study did not investigate the timing of SRS with regards to systemic therapy. Miller at al reported on a large retrospective study of 547 patients presenting with 3224 brain metastases treated with radiotherapy and targeted therapies and found that concurrent HER-2/epidermal growth factor receptor tyrosine kinase inhibitors with gamma knife SRS significantly decreased 12-month cumulative incidence of local failure from 15.1% to 5.7% (P<0.001) (41). Similarly, they found that concurrent HER-2 antibody treatment with concurrent SRS decreased 12-month cumulative incidence of local failure from 18.4% to 10.2% (P = 0.003), demonstrating synergy with use of concurrent SRS and HER2/EGFR TKIs and HER-2 antibody therapies. Unfortunately, the same synergy was not found in hormone receptor positive breast cancer patients with brain metastases treated with concurrent hormone therapy and SRS.

Better blood-brain barrier penetrating small tyrosine kinase inhibitors were subsequently developed after first generation trastuzumab and include lapatinib, afatinib, epertanib, neratinib tucatinib, pyrotinib and are used as systemic targeted therapy for patients with HER-2 positive breast cancer (53). Lapatinib was one of the first small molecule dual tyrosine kinase inhibitors targeted against EGFR 1 and HER-2 pathways. Kim et al. reported on 84 patients with 487 HER-2 amplified breast cancer brain metastases and treatment with SRS alone versus concurrent SRS and lapatinib and found that patients with concurrent therapy had higher rates of complete response (35% versus 11%, p = 0.008) (42). Furthermore, best per-lesion objective response was superior in the concurrent lapatinib group with a median 100% objective response versus 70% reduction (p < 0.001). This group did not find an increased risk of grade 2 radiation necrosis with concurrent therapy. However, an interesting finding of the study was that lapatinib did not have protective effects on distant intracranial failure rates; one of the main avenues in which concurrent SRS and systemic therapies were thought to theoretically improve overall survival.

Parsai et al. recently reported on 126 patients with HER-2 positive breast cancer with 479 brain metastases; 24 patients received concurrent treatment with SRS and lapatinib. They found SRS with concurrent lapatinib was associated with reduction in local failure at 12 months reported as 5.7% compared to 15.1% in the nonconcurrent therapy group (P < 0.01) (43). Local failure decreased for lesions less than or equal to 75^th^ percentile by volume but did not have a significantly improved local failure rate for lesions greater than the 75^th^ percentile. Furthermore, any use of lapatinib after development of brain metastases improved median survival compared to SRS alone (27.3 months versus 19.5 months, p = 0.03). Unlike the Kim et al. study, this supports the theory that targeted therapies may improve overall survival by controlling distant intracranial failure and systemic extracranial disease.

The majority of breast cancers are hormone receptor (HR) positive with endocrine therapy being the mainstay of systemic therapy and including antiestrogen therapy with selective estrogen receptor modulators, aromatase inhibitors, and/or selective estrogen receptor (ER) degraders and combination with cyclin-dependent kinase 4/6 inhibitors (54). Unfortunately, 15 to 20% of ER positive breast cancers are intrinsically resistant to endocrine therapy and another 30 to 40% develop resistance after treatment (55, 56). One of the described escape mechanisms contributing to hormone resistance involves activation of the second depending kinase 4 and 6 pathways in the presence of hormone receptor antagonists (44, 57). A study by Mills et al. reported HR positive breast cancers patients with hormonal therapy prior to stereotactic radiotherapy (SRT) report 2-year overall survival as low as 24% (58), however there is little literature investigating concurrent SRS with systemic endocrine therapy.

Figura et al. report on a retrospective study involving 15 patients and 42 lesions in patients with HR positive brain metastases treated with SRS or fractionated stereotactic radiotherapy (FSRT) within 6 months of CDK 4/6 inhibitor administration. Radiotherapy was delivered concurrently, before, or after CDK4/6 inhibitors in 18 (43%), 9 (21%), and 15 (36%) lesions, respectively (44). Fourteen percent of the cohort received CDK inhibition alone, 48% of the cohort CDK inhibition plus fulvestrant and 38% CDK inhibition plus an aromatase inhibitor. 6- and 12-month local control of treated lesions were reported as 88% and 88%, respectively, while 6- and 12-month distant brain control was 61% and 39%, respectively, with median overall survival of 36.7 months from diagnosis of brain metastases (44). A significant portion of this cohort received concurrent therapies and the median overall survival was much higher at 36.7 months than the 13.3 month median overall survival recently reported in a 2021 ASCO meeting abstract by Wang et al. in patients who received SRS upfront for treatment of HR+/HER-2 negative breast cancer brain metastases (59). This led the Figura group to conclude that SRT to breast cancer brain metastases is well-tolerated without significant increase in neurotoxicity when combined with CDK 4/6 inhibitors, and although brain metastases control rates were similar to prior historical data, there was a synergy between SRT and the systemic therapy which prolonged median overall survival.

### Melanoma

Approximately 99,780 patients will be diagnosed with melanoma in the United States in 2022 (60), with nearly half developing brain metastases over the course of their disease (61). Approximately 40 to 60% of cutaneous melanoma patients have BRAF mutations which results in constitutive activation of BRAF, and downstream mitogen activated protein kinase (MAPK) pathway (62, 63). For this reason, many of the targeted therapies used in the treatment of metastatic melanoma include BRAF inhibitors such as dabrafenib, vemurafenib, encorafenib, and MEK1/2 inhibitors such as binimetinib (53).

A 2015 study by Ahmed at al evaluating LINAC-based SRS with concurrent vemurafenib found that patients had a median overall survival from the date of SRS of 7.2 months with a median survival from date of brain metastases diagnosis of 11.9 months (64). In this study, therapies were truly concurrent, with vemurafenib being held only 2 to 3 days pre- and post-SRS treatment. It did not show any evidence of increased toxicity with a combination of SRS and targeted therapy and concluded that concurrent therapy appeared to be safe and effective. Subsequent studies have further investigated and similarly reported synergy between SRS and targeted therapies in patients with melanoma brain metastases (**Table 3**).

**Table 3:** Studies evaluating synergy between SRS and targeted therapies in patients with melanoma brain metastases (BrM).

Study Identifier	Study Period	Study Size (n = patients)	Treatment/Intervention Groups	Results	References
Ahmed et al.	2010-2013	N = 24	Patients with metastatic melanoma BrM treated with SRS while on vemurafenib	• Fourteen (58%) patients had distant brain failure at a median of 3.4 months • Median OS from the date of SRS = 7.2 months (range 1.5–26.8 months) • Median OS from date of BrM diagnosis = 11.9 months (range 1.5–28.5 months) • No evidence of increased toxicity with concurrent SRS + vemurafenib	(64)
Xu et al.	2010-2014	N = 65	Patients with metastatic melanoma BrM treated with SRS +/- BRAF inhibitors	• Median OS after diagnosis of BrM and after SRS were favorable in patients with BRAF mutation and treated with SRS + BRAFi (23 months and 13, respectively, p < 0.01) • SRS local tumor control rate of 89.4% in the entire cohort • Local control rate improved in the patients treated with SRS + BRAFi compared to BRAF mutated patients without BRAFi treatment and wild-type patients	(65)
Ahmed et al.	2007-2015	N = 96	Patients with metastatic melanoma BrM treated with single-session SRS and anti-PD-1 therapy, anti-CTLA-4 therapy, BRAF/MEK inhibitors(i), BRAFi, or conventional chemotherapy	• 12-month distant control rates = 38%, 21%, 20%, 8%, and 5% (P = 0.008) for SRS with anti-PD-1 therapies, anti-CTLA-4 therapy, BRAF/MEKi, BRAFi, and conventional chemotherapy, respectively. • No significant differences in local control rates • Treatment with anti-PD-1 therapy, anti-CTLA-4 therapy, or BRAF/MEKi significantly improved OS on both univariate and multivariate analyses when compared with conventional chemotherapy	(66)
Kotecha et al.	1987-2014	N = 366	Patients with metastatic melanoma BrM treated with SRS + targeted and immunotherapies	• On MVA, younger age, lack of extracranial mets, better KPS, and fewer BrM, and treatment with BRAF inhibitors, anti–PD-1/CTLA-4 therapy, or cytokine therapy were significantly associated with OS • For patients who underwent SRS, the 12-month LF rate was lower among those with BRAFm lesions *vs* BRAFwt lesions (6% *vs* 22%, p < 0.01) • 12-month LF rates among lesions treated with BRAFi and PD-1/CTLA-4 agents were 1% and 7%, respectively • On MVA, BRAF inhibition within 30 days of SRS was protective against LF (HR 0.08, 95% CI 0.01–0.55; p = 0.01) • 12-month rates of RN were low among lesions treated with BRAFi (0%), PD-1/CTLA-4 inhibitors (2%) and cytokine therapies (6%)	(67)
Murphy et al.	2011-2017	N = 26	Patients with metastatic melanoma BrM treated using pembrolizumab, nivolumab and/or ipilimumab, sequentially, or concurrently with SRS	• Median OS = 26.1 months • Following concurrent SRS and immunotherapy, patients had a significantly longer period of intracranial progression free survival than those treated with nonconcurrent therapy, 19 months versus 3.4 months (P < 0.0001) • No grade 4-5 toxicities were observed	(68)
Mastorakos et al.	2011-2015	N = 198	Patients with metastatic melanoma BrM treated with SRS +/- BRAF kinase inhibitors	• On MVA, BRAF mutation was an independent, positive prognostic factor with a hazard ratio of 0.59 • BRAF mutated patients who received BRAFi following SRS had improved survival compared to those who received it before (P < .001) or concurrently (P = .007) • PD-1 inhibitors improved survival, with more pronounced effect in patients not carrying the BRAF mutation • Among the patients treated with BRAFi, 10.4% developed intracerebral hematoma (ICH), in comparison to 3% of patients not treated with BRAFi (P = .03)	(69)
Schaule et al.	2011-2018	N = 110	Patients with metastatic melanoma BrM treated with targeted therapies or immunotherapy and concurrent (≤30 days) SRT	• Median OS = 8.4 months • Cumulative BrM volume, timing of metastases (syn- *vs*. metachronous) and systemic therapy with concurrent IT influenced OS significantly • Based on these parameters, the VTS (volume-timing-systemic therapy) score was established and stratified patients into three groups with a median OS of 5.1, 18.9 and 34.5 months, respectively (p < 0.05)	(70)
Wang et al.	2007-2019	N = 431	Patients with metastatic melanoma BrM treated with various local and systemic therapies	• Mucosal subtype (p = 0.022), LDH level (p = 0.005), no extracranial metastasis (p = 0.01), concurrent liver metastasis (p = 0.004), local treatment (p = 0.001) and use of PD-1 inhibitors (p < 0.0001) were independent prognostic factors for OS • Mucosal subtype BrM had poor response to PD-1 inhibitors (p = 0.007), with a shorter intracranial PFS than other subtypes • In patients with BRAF mutated melanoma BrM, first-line BRAF/MEK inhibitor therapy had an advantage in OS compared to the first-line anti-PD-1 therapy group (p = 0.043)	(71)
Wegner et al.	2010-2015	N = 247	Patients with metastatic melanoma BrM treated with immunotherapy and SRS	• Immunotherapy prior to SRS, within 0–7 days of SRS, and greater than 7 days from SRS had 3-year survival rates of 21%, 55%, and 35%, respectively (p = 0.0153) • Multivariable Cox regression identified lack of extracranial disease, more recent year of treatment, and time from SRS to immunotherapy of 0–7 days as predictors of improved survival	(72)
Khan et al.	2010 meta-analysis	N = 976	Searched for studies comparing patients with metastatic melanoma BrM treated with SRS +/- BRAF inhibitors	• Survival significantly improved for patients receiving BRAF inhibitor plus SRS *vs* SRS alone as assessed from the time of SRS induction (p < 0.00001), from the time of brain metastasis diagnosis (p < 0.00001), or from the time of primary diagnosis (p = 0.02) • Dual therapy was also associated with improved local control (p = 0.03) • Intracranial hemorrhage was higher in patients receiving BRAF inhibitors plus SRS than in those receiving SRS alone (p = 0.004)	(73)

Xu et al. subsequently evaluated use of BRAF kinase inhibitors in conjunction with SRS for patients with melanoma brain metastases and found that patients with BRAF mutations treated with BRAF inhibitors had improved median survival times from diagnosis, and after SRS, of 23 months and 13 months (p < 0.01), respectively (65). This was statistically significant compared to the BRAF wild-type group. In conjunction with SRS, they reported a local control rate of 92% at 1 year in patients with BRAF mutations treated with BRAF inhibitors, compared to 82.4% in patients with BRAF mutations not treated with BRAF inhibitors and 69.2% in patients who were BRAF wild-type.

A 2016 study by Ahmed at al investigating clinical outcomes in patients with melanoma brain metastases treated with SRS and anti-PD1, anti-CTLA4, BRAF/MEK inhibitors, BRAF inhibitors, and conventional therapy found distant 1-year disease control rate of 20% and 8% for BRAF/MEK inhibitors and BRAF inhibitors, respectively, and significantly improved overall survival for patients treated with anti-PD1, anti-CTLA4 and BRAF/MEK inhibitors when compared to those treated with conventional chemotherapy (66). This study is important because it demonstrated that targeted therapies and immunotherapies synergistically contribute to SRS by helping improve distant brain metastases control rates.

An important paper demonstrating synergy between multiple therapies is by Kotecha et al, in which 366 patients were treated for 1336 melanoma brain metastases. They found that younger age, lack of extracranial metastases, better Karnofsky performance status score, fewer melanoma brain metastases, as well as treatment with BRAF inhibitors, anti-PD1/CTLA4 therapies, or cytokine therapy were significantly associated with improved overall survival (67). Among patients who underwent SRS, patients with BRAF mutant lesions had a 12-month local failure rate of 6% compared to 22% and BRAF wild-type patients. Furthermore, 12-month local failure rates in patients treated with BRAF inhibitors and PD1/CTLA-4 agents were 1% and 7%, respectively. On multivariate analysis, BRAF inhibition within 30 days of SRS was protective against local failure (p = 0.01); 12-month radiation necrosis rates were 0% in patients treated with BRAF inhibitors, 2% in patients treated with PD1/CTLA-4 inhibitors, and 6% of patients treated with cytokine therapies.

Similarly, Murphy et al. found that following concurrent SRS and immunotherapy within 30 days, patients had significantly longer period of intracranial progression free survival than those treated without concurrent therapy, 19 months versus 3.4 months (P < 0.0001), with no grade 4-5 toxicities observed (68). A multicenter retrospective study by Mastorakos et al. evaluated patients with BRAF-mutated melanoma brain metastases and BRAF kinase inhibitor use in conjunction with SRS and found that BRAF-mutated patients who received BRAF inhibitors following SRS had improved survival compared to patients who received it before (p<0.001) or concurrently (p = 0.007) (69). This study supports synergy between use of targeted therapies and SRS but highlighted the importance of their timing in order to maximize clinical benefit.

Schaule et al. conducted a retrospective analysis of 110 patients treated with concurrent targeted or immunotherapy and stereotactic radiotherapy and found that cumulative brain metastases volume (p = 0.04), timing of metastases (syn-versus metachronous) (p = 0.01) and systemic therapy with concurrent immunotherapy (p = 0.005) significantly improved overall survival; with these findings they established a volume-timing-systemic therapy (VATS) score with point values ascribed to the aforementioned factors and median overall survival as of 34.5 months in patients with a VATS score of 2 (p = 0.03) (70).

With multiple studies demonstrating synergy between SRS and systemic therapies, Wang et al. sought to identify clinicopathologic characteristics and prognostic factors in patients with melanoma brain metastases. They found that in patients with BRAF-mutated melanoma brain metastases, first-line treatment with BRAF/MEK inhibitor therapy improved overall survival compared to patients treated with first-line therapy with anti-PD1 (P = 0.043) (71). This is the first study in the literature promoting BRAF/MEK inhibitors as a superior first-line therapy in patient with BRAF-mutated melanoma brain metastases. Although it did not specifically seek to elucidate synergy with SRS, 49% of this cohort also received SRS.

A 2021 study by Wegner et al. concluded that immunotherapy within 7 days of SRS had a statistically significant association with improved outcomes and 3-year survival rate of 55% (P equals 0.0153) (72). This study also illustrating that the timing of systemic therapy with relation to SRS delivery may affect clinical outcomes. Lastly, a 2021 meta-analysis including 8 studies and involving 976 patients with melanoma brain metastases found that dual therapy of BRAF inhibitors in combination with SRS improved survival (P < 0.00001) and local control (P = 0.03), further supporting the literature of synergy between these two therapies (73).

### Renal Cell Carcinoma

Approximately 320,000 patients are diagnosed with renal cell carcinoma (RCC) worldwide (74), with 10% to 16% developing brain metastases (75, 76). Eighty percent of all renal cell carcinoma cases are clear-cell type and 90% of these develop a von-Hippel-Lindau tumor suppressor gene mutation that leads to activation of multiple genes including vascular endothelial growth factor (VEGF) with subsequent angiogenesis being a primary mechanism of progression in advanced RCC (77). For this reason, targeted therapies against VEGF-tyrosine kinases are included as part of first-line therapy for metastatic renal cell carcinoma. VEGF-tyrosine kinase inhibitors include sunitinib, pazopanib, and sorafenib; with newer multi-targeted tyrosine kinase inhibitors such as cabozantinib, which inhibits VEGFR/MET/AXL (78), and lenvatinib, which inhibits VEGFR 1, 2, 3/FGFR1, 2, 3, 4/PDGFR alpha/RET/KIT (79). Many studies assessing the synergy between SRS and targeted therapies utilize these agents (**Table 4**). Other targeted therapies including mammalian target of rapamycin complex 1 (mTORC1) inhibitors and immune checkpoint inhibitors, including anti-programmed death receptor 1 (PD-1) and anti-cytotoxic T-lymphocyte associated protein 4 (CTLA-4) monoclonal antibodies, have also been used to treat and improve overall survival in patients with extracranial metastatic renal cell carcinoma (87–91).

**Table 4:** Studies evaluating synergy between SRS and targeted therapies in patients with renal cell carcinoma brain metastases (BrM).

Study Identifier	Study Period	Study Size (n = patients)	Treatment/Intervention Groups	Results	References
Cochran et al.	1999-2010	N = 61	Patients with metastatic renal cell carcinoma BrM treated with Gamma Knife surgery and targeted agents such as tyrosine kinase inhibitors, mammalian target of rapamycin inhibitors, and bevacizumab	• Median survival for patients receiving targeted agents was 16.6 months compared with 7.2 months for those not receiving targeted therapy (p = 0.04). • Freedom from local failure at 1 year was 93% versus 60% for patients receiving and those not receiving targeted agents, respectively (p = 0.01) • MVA showed use of targeted agents (hazard ratio 3.02, p = 0.003) was the only factor that predicted for improved survival	(80)
Vickers et al.	2005-2011	N = 106	Patients with metastatic renal cell carcinoma BrM treated with targeted therapies; 77 patients were treated with sunitinib, 23 patients with sorafenib, 5 with bevacizumab, and 1 with temsirolimus. 81.1% received WBRT and 24.8% received SRS	• On MVA, KPS < 80%, diagnosis to treatment with targeted therapy < 1 year, and a higher number of BrM (>4) was associated with worse survival from time of diagnosis with BrM • Patients diagnosed with BrM at the initiation of targeted therapy had a survival of 19.1 months while patients who developed BrM while receiving targeted therapy had a survival of 6.3 months	(81)
Bates et al.	2003-2014	N = 25	Patients with metastatic renal cell carcinoma BrM who received WBRT, SRS, or both; 28% of patients were receiving a concurrent kinase inhibitors (KI) at the time of radiotherapy	• No significant difference in overall survival or brain progression free survival (BPFS) for SRS compared with WBRT or WBRT and SRS combined • Concurrent use of KI was not associated with any change in OS or BPFS	(82)
Barata et al.	2005-2017	N = 95	Patients with metastatic clear cell renal carcinoma BrM treated with SRS and stratified by changing or continuing systemic treatment (VEGFR tyrosine kinase inhibitors, mTOR inhibitors, immune checkpoint, or other therapies)	• Local control with SRS was achieved in 85% of the patients • Most common systemic treatment at SRS included anti-vascular endothelial growth factor (67%), mammalian target of rapamycin (14%), and programmed cell death protein 1 inhibitors (9%) • No difference in median overall survival was found for the STAY and SWITCH groups (24.2 *vs*. 27.1 months; p = .381) but was significantly longer than patients with progression outside of the SRS sites who switched systemic therapy (8.5 months; p = .025)	(83)
Sperduto et al.	2006-2015	N = 711	Patients with metastatic renal cell carcinoma with new BrM treated with various regimens of radiotherapy/targeted therapies	• Median survival 12 months • Four prognostic factors (Karnofsky performance status, extracranial metastases, number of BrM, and hemoglobin b) were significant for survival after the diagnosis of BrM • Of the 6 drug types studied, only cytokine use after BrM was associated with improved survival • Use of WBRT declined from 50% to 22%, and the use of SRS increased from 46% to 58%	(77)
Juloori et al.	1998-2015	N = 367	Patients with metastatic renal cell carcinoma BrM treated with various regimens of radiotherapy/targeted therapies	• Median OS was 9.7 months • KPS and number of BrM were the only factors prognostic for OS • 147 patients (39%) received VEGFR tyrosine kinase inhibitors (TKIs) • Median OS was significantly greater among patients receiving TKIs (16.8 *vs* 7.3 months, p < 0.001) • On MVA, KPS, number of metastases, and TKI use remained significantly associated with OS • TKIs did not significantly decrease the 12-month cumulative incidence of local failure (11.4% *vs* 14.5%, p = 0.11) • On MVA, age, number of BrM, and lesion size remained associated with local failure • 12-month cumulative incidence of radiation necrosis was 8.0%; use of TKIs within 30 days of SRS was associated with a significantly increased 12-month • Cumulative incidence of radiation necrosis (10.9% *vs* 6.4%, p = 0.04)	(84)
Khan et al.	2020 systematic review and meta-analysis	N = 897	Studies comparing TKIs in combination with SRS to SRS alone for treatment of patients with metastatic renal cell carcinoma BrM	• TKI use associated with better survival (HR 0.60 [0.52, 0.69], p < 0.00001) and local brain control (HR 0.34 [0.11, 0.98], p = 0.05) • SRS subgroup revealed significantly better survival (HR 0.61 [0.44, 0.83], p = 0.002) and local brain control (HR 0.19 [0.08, 0.45], p = 0.0002)	(85)
Stenman et al.	2005-2014	N = 43	Patients with metastatic renal cell carcinoma BrM treated with single-fraction gamma knife radiosurgery (sf-GKRS) in era of targeted agents (TA) and immune checkpoint inhibitors	• LC rates at 12 and 18 months were 97% and 90%, respectively • Median OS from the first sf-GKRS was 15.7months • Low serum albumin (HR for death 5.3), corticosteroid use pre-sf-GKRS (HR for death 5.8) and KPS < 80 (HR for death 9.1) were independently associated with worse OS • Adverse radiation effects (ARE) were seldom symptomatic and were associated with tumor volume, 10- Gy volume and pre-treatment perifocal edema • ARE were less common among patients treated with TA within 1 month of sf- GKRS	(86)

When reviewing outcomes for 61 patients with renal cell carcinoma brain metastases treated with targeted agents and gamma knife radiosurgery, Cochran et al. showed that the median survival for patients receiving targeted agents was 16.6 months compared to 7.2 months, with freedom from local failure at 1 year being 93% versus 60% (p = 0.01) (80). Their multivariate analysis also showed that utilization of targeted therapies was the only factor that predicted improved survival. Subsequently, Vickers et al. assessed prognostic factors for survival in patients with RCC brain metastases treated with targeted therapies and found KPS less than 80, diagnosis to treatment with targeted therapy less than a year, and greater than 4 brain metastases were associated with worse survival (81). In this study, 81.1% received whole brain radiotherapy (WBRT) and 24.8% received SRS and they found that patients diagnosed with brain metastases at the initiation of targeted therapy had a survival of 19.1 months, while patients who developed brain metastases while receiving targeted therapy had a survival of 6.3 months.

Bates et al. reviewed 25 consecutive patients who received radiotherapy consisting of WBRT and SRS in addition to targeted therapies and found no significant difference in overall survival or brain progression free survival with concurrent use of kinase inhibitors and radiotherapy (82). Although not statistically significant, there was a trend towards improved median overall survival in patients treated with concurrent kinase inhibitors compared to those not treated with concurrent kinase inhibitors, 7.3 months versus 4.1 months, respectively. Furthermore, this study only included first generation kinase inhibitors (sunitinib, sorafenib, or pazopanib) and not newer multi-targeted kinase inhibitors. Subsequent to these findings, Barata et al. reported on the effect of switching systemic treatment after SRS for oligoprogressive metastatic renal cell carcinoma and found no difference in median overall survival between patients who remain on the same systemic therapy and those who switched to another systemic therapy after SRS for their progressive disease (83). Those who remained on the same systemic therapy had a median overall survival of 24.2 months and those were switched 27.1 months (p = 0.381). Patients with progression outside of the SRS sites who switched systemic therapy had a significantly worse overall survival of 8.5 months. The systemic therapies in this retrospective study included 67% anti-VEGF inhibitors, 14% mTOR1 inhibitors, and 9% program cell death protein 1 inhibitors. Furthermore, although not the main endpoint of the study, median overall survival of patients who switched systemic therapies after SRS for oligoprogressive renal cell brain metastases was 27.1 months, which was an improvement in previously reported overall survival and illustrated the positive effects of a multimodal, multidisciplinary approach to improving outcomes for patients with oligoprogressive disease.

In a large multi-institutional retrospective study assessing 711 renal cell carcinoma patients with new brain metastases, prognostic factors affecting survival included Karnofsky performance status, extracranial metastases, number of brain metastases, and hemoglobin; only cytokine use after brain metastases was associated with improved survival (77). Conversely, initiation of VEGF targeted TKI, mTOR targeted TKI, immunotherapy, antiangiogenic drugs, and cytotoxic chemotherapy prior to diagnosis of brain metastases was associated with greater risk of death. Although demonstrating benefit for cytokine use, it is important to note that newer multi-target tyrosine kinase inhibitors were likely excluded from this study, given that the study recruited participants up until 2015 and many of the newer multi-target tyrosine kinase inhibitors were subsequently developed.

In 2020, Juloori et al. reported on overall survival and response to radiation and targeted therapy in 367 patients with 912 renal cell brain metastases. They found that median overall survival was significantly greater among patients receiving TKI’s (16.8 versus 7.3 months, p < 0.001) and that TKI use was significantly associated with improved overall survival after multivariate analysis (84). Similarly, a meta-analysis by Khan et al. evaluating the impact of TKI use combined with radiation therapy (n= 897) found that TKI use was associated with better survival (HR 0.60 [0.52, 0.69], p < 0.00001) and local control (HR 0.34 [0.11, 0.98], p = 0.05), although it did not affect distant brain control and brain progression free survival (85).

Finally, the most recent study by Stenman et al. in 2021 evaluated 43 patients with 194 targets that were irradiated with 88% of the cohort also receiving targeted therapies. This cohort was treated with single fraction gamma knife radiosurgery (sf-GKRS) after a median time of 8.5 months from metastatic renal cell carcinoma diagnosis and subsequent to sf-GKRS had a median overall survival of 15.7 months; reflecting a cumulative median overall survival of 24.2 months (86). Although the study did not show targeted agents to be associated with improved survival, when compared to historical data, a median overall survival at 24.2 months is an improvement in overall survival and supports the existence of synergy between targeted therapies and SRS. However, optimal administration timing of these therapies and physiologic explanation of their interaction remains to be elucidated.

## Discussion

Synergy between SRS and targeted therapies has been demonstrated and found to improve outcomes for patients with non-small cell lung, HER-2 positive and endocrine receptor positive breast, melanoma, and renal cell carcinoma brain metastases. The varied pathophysiological mechanisms behind radiation-induced synergy are beyond the scope of this review; they include but are not limited to: increase in expression of major histocompatibility complex class I, calreticulin, and Fas cell surface death receptor, release of high mobility group box 1 nuclear protein, activation of dendritic cells and enhanced tumor antigen cross-presentation, increase in tumor-infiltrating lymphocyte density, and modulation of immune checkpoint molecule expression and regulatory T cells (92). Improvement in overall survival for patients with these diagnoses has only been made possible *via* a multidisciplinary approach between medical oncologists, radiation oncologists, and neurosurgeons. Several difficulties exist when trying to compare results from multiple studies assessing outcomes in patients with brain metastases. One challenge is that primary and secondary endpoints between studies vary, with some studies evaluating certain variables and other studies evaluating others. Some examples of primary and secondary endpoints include overall survival, rates of radiation necrosis, local and distant brain metastases control, and neurotoxicity. Standardization of primary and secondary endpoints would help to better compare outcomes of future studies. Another challenge with assessing the results of studies evaluating concurrent SRS with targeted therapy use is that the definition of “concurrent” also varies from study to study. Some studies define “concurrent” as actively on systemic therapy, others with systemic therapy only held for 1 to 2 days before and after SRS, while other studies define concurrent therapy as having occurred with the initiation of targeted therapy within 30 days, 3 months, 6 months or even within 12 months before or after SRS.

A final challenge is that the definition of synergy varies and that there are different types of synergy. One type of synergy involves additive, enhancing synergy from concurrent therapies in which the combined effects of two therapies contribute to a greater, durable clinical effect compared to if the individual therapies were applied alone and/or in sequential order at varying time points. This type of synergy has not been clearly supported in the literature. Another type of synergy involves cumulative synergy in which various therapies are applied at various time points to maximize therapeutic effects, increase progression free survival, overall survival, and quality of life for patients with brain metastases. Cumulative synergy is difficult to study given that comprehensive cancer care is tailored to each individual patient and that varied treatments may be implemented at varied timepoints depending on a patient’s clinical status. Evaluation of cumulative synergy involves evaluation of treatment paradigms in their totality rather than a discrete response to a single treatment. The literature assessing cumulative synergy is lacking. An example of cumulative synergy was reported by Cristaudo et al. in which they described the clinical course of a patient with metastatic melanoma and 10 brain metastases which were treated with SRS with complete response (including untreated lesions), was then started on BRAF/MEK inhibitors and subsequently required other treatment modalities as new metastases occurred (93). Eight months after her initial SRS treatment she developed new metastases which responded to SRS once again and 7 months later had new lesions treated with whole brain radiotherapy and was started on immunotherapy. Twenty months after initial diagnosis the patient had a Karnofsky performance score of 100 with no radiologic signs of toxicity. This report demonstrates how therapies can exhibit a cumulative synergy and underscores the importance of a multidisciplinary approach in the treatment of patients with brain metastases. There is unlikely to be a one-time combination of therapies that will achieve permanent local and distant disease control. However, it is the cumulative synergy of various treatment modalities, used at various timepoints in clinical disease and in various combinations, that will likely lead to the most clinical benefit.

Future clinical trials with standardized inclusion criteria and shared endpoints are needed to better elucidate concurrent and cumulative synergy between SRS and targeted therapies in the treatment of patients with brain metastases. Newer targeted therapies proven to impact time to CNS progression and/or progression free survival must continue to be investigated for synergy with SRS. These future studies must include patients with active, symptomatic metastases and avoid over-recruitment of clinically silent, stable metastases. While important for future studies to focus on critical endpoints such as overall survival, response rate, and local control, it is equally important that they focus on understanding the toxicity associated with combination therapies. Additional basic science research is also needed to better understand brain metastases’ molecular profiles, how they relate to their primary solid tumors, and how they may change after treatment with targeted therapy and radiotherapy. Lastly, multi-institutional collaboration is needed to achieve larger sample sizes, better external validity, faster accrual and, hopefully, more meaningful, positive results.

## Author Contributions

AE contributed to the conception of the manuscript. SR and AE contributed to the design of the manuscript. SR contributed to the data acquisition, assessment, and review; creation of initial manuscript/tables; and critical review and revision of the manuscript. DO, NT, MV, PF, HY, KA, and AE contributed to the critical review and revision of the manuscript. All authors contributed to the article and approved the submitted version.

## Conflict of Interest

The authors declare that the research was conducted in the absence of any commercial or financial relationships that could be construed as a potential conflict of interest.

## Publisher’s Note

All claims expressed in this article are solely those of the authors and do not necessarily represent those of their affiliated organizations, or those of the publisher, the editors and the reviewers. Any product that may be evaluated in this article, or claim that may be made by its manufacturer, is not guaranteed or endorsed by the publisher.
